# Effectiveness of Elastomeric Half-Mask Respirators vs N95 Filtering Facepiece Respirators During Simulated Resuscitation: A Nonrandomized Controlled Trial

**DOI:** 10.1001/jamanetworkopen.2021.1564

**Published:** 2021-03-16

**Authors:** Andrew J. Barros, Costi D. Sifri, Taison D. Bell, Joshua C. Eby, Kyle B. Enfield

**Affiliations:** 1 Division of Pulmonary and Critical Care Medicine, Department of Medicine, University of Virginia School of Medicine, Charlottesville; 2 Division of Infectious Disease and International Health, Department of Medicine, University of Virginia, School of Medicine, Charlottesville; 3 Office of Hospital Epidemiology/Infection Prevention & Control, UVA Health, Charlottesville, Virginia; 4 Employee Health, UVA Health, Charlottesville, Virginia

## Abstract

This nonrandomized controlled trial compares the effectiveness of elastomeric half-mask respirators with that of N95 filtering facepiece respirators during cardiopulmonary resuscitation.

## Introduction

Respirators provide protection from airborne particles, and the N95 filtering facepiece respirator (FFR) is the most commonly used type in health care. Recent FFR shortages have led to increased interest in reusable elastomeric half-mask respirators (EHMRs),^1,2^ which have a flexible interface and larger straps than FFRs. Data from simulation and industrial settings suggests that EMHRs may provide higher respiratory protection than FFRs.^3^ Case reports in previous severe acute respiratory syndrome coronavirus pandemics have suggested transmission during cardiopulmonary resuscitation (CPR) despite FFR use,^4^ and simulation studies have demonstrated an unacceptable leak during CPR in up to 40% FFR users.^5^ Owing to shortages of FFR models, individuals who could not be fit in an available FFR model were fit for an EMHR. We assessed whether EHMRs provide improved fit during simulated CPR compared with FFRs.

## Methods

We conducted a nonrandomized controlled trial of clinicians and health care workers (physicians, advanced practice clinicians, nurses, and nursing assistants) working at a single institution from October 26, 2020, to November 4, 2020 (NCT04591756). The University of Virginia Institutional Review Board for Health Sciences Research approved this study, and all participants provided verbal informed consent. The Transparent Reporting of Evaluations With Nonrandomized Designs (TREND) reporting guideline was used for this study.^6^

Participants were recruited from wards that cared for patients with coronavirus disease 2019 (COVID-19). All participants had previously completed a National Institute for Occupational Safety and Health–approved fit test, had been assigned a model of EHMR or FFR for use, and had used recently their assigned model of respirator. At our institution, disposable FFRs are used for a single patient care session, captured for inspection and reprocessing, returned for reuse up to 6 times, and then discarded.

Each participant preformed chest compressions on a mannequin while denatonium benzoate was aerosolized into a fit-testing hood (trial protocol in Supplement 1) and while wearing their assigned model of EHMR or FFR. Testing was stopped after 2 minutes or when the participant reported detection of the agent. Our primary end point was detection of the test agent indicating poor respiratory fit. The secondary end point was the time to detection of the agent. Information on employment role, years of respirator use, and respirator model was collected.

We hypothesized that FFRs would have a 40% failure rate and that EMHR would provide a 50% relative risk reduction. We calculated that 81 participants per group would give 80% power with a 5% type I error rate. Interim analyses of the primary end point were preplanned with 50, 100, and 150 participants using the Haybittle-Peto boundary (ie, 2-sided *P* < .001 was considered significant for early stopping and 2-sided *P* < .05 for final analysis). The χ^2^ test was used for all count data. Analyses were performed using R, version 4.0.2 (R Foundation for Statistical Computing).

## Results

The final analysis included 100 participants (Table). The study was stopped after the second interim analysis crossed the prespecified threshold. Participants reported detection of the agent in 0 of 36 tests in the EMHR group and 18 of 64 tests (28.1%) in the FFR group (risk difference, −28.1%; 95% CI −39.1% to −17.1%). Participants reported detection of the agent at a median of 69 seconds (interquartile range, 42-107 seconds) (Figure). An association between the primary end point and participant employment role, years of respirator use, or FFR model was not found.

**Table.** Participant Characteristics and Results

	Participants, No. (%)	
	N95 filtering facepiece respirator (n = 64)	Elastomeric half mask respirator with P100 filters (n = 36)
Provider role		
MD, DO, NP, or PA	6 (9)	5 (14)
RN, RT, PT, or OT	56 (88)	29 (81)
Unlicensed staff	3 (5)	1 (3)
Duration of respiratory protection use, y		
<5	26 (41)	12 (33)
5 to <10	21 (33)	18 (50)
10 to <15	9 (14)	1 (3)
>15	8 (13)	5 (14)
Respirator model used		
3M 1860	11 (17)	NA
3M 1860S	19 (30)	NA
Dasheng DTC3B	6 (9)	NA
Halyard 46767	22 (34)	NA
Halyard 46867	6 (9)	NA
3M 6300 with P100 filters	NA	36 (100)
Sensitivity check result, squeezes		
10	62 (97)	36 (100)
20	2 (3)	0
30	0	0
Agent detected		
No	46 (72)	36 (100)
Yes	18 (28)	0
Time to agent detection (IQR), s	69 (42-90)	NA

Abbreviations: DO, doctor of osteopathic medicine; IQR, interquartile range; MD, doctor of medicine; NA, not applicable; NP, nurse practitioner; OT, occupational therapist; PA, physician assistant; PT, physical therapist; RN, registered nurse; RT, respiratory therapist.

**Figure.**
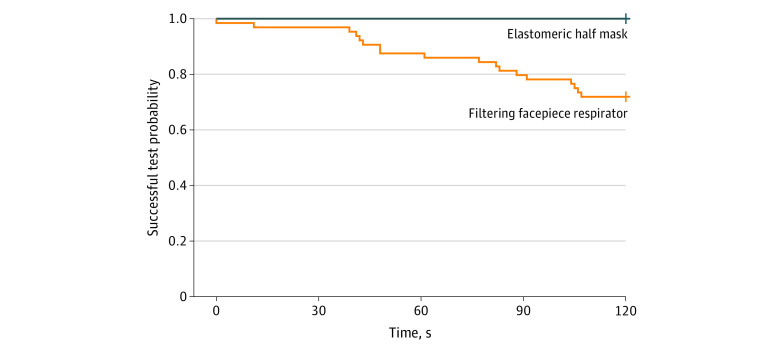
Time to Test Failure by Mask Type

## Discussion

Our results suggest that FFR fit during CPR is poor and that EMHRs provide superior fit, confirming previous research.^5^ We chose to evaluate fit during CPR because it is highly aerosolizing, physically strenuous, and has been associated with occupational transmission. Strengths of our study include our real-world design and prospective data collection. Limitations include the lack of blinding or randomization and the use of participant-reported detection. The data suggest that the EMHR is more effective at preventing aerosol inhalation during strenuous clinical work and should be considered for preventing COVID-19 transmission.

## Notes

Supplement 1. **Trial Protocol**

Supplement 2. **Data Sharing Statement**
