# Supplementary material for: Effectiveness of Elastomeric Half-Mask Respirators vs N95 Filtering Facepiece Respirators During Simulated Resuscitation: A Nonrandomized Controlled Trial
Source: JAMA Netw Open. 2021 Mar 16;4(3):e211564. doi: 10.1001/jamanetworkopen.2021.1564 (PMC7967080; doi:10.1001/jamanetworkopen.2021.1564)
Supplement: Supplement 1. — Trial Protocol [file jamanetwopen-e211564-s001.pdf]

Supplementary Appendix: Protocol

Required Materials:

- A test enclosure, similar to 3M hood assembly parts #FT 14 and #FT15 combined.
- DeVilbiss Model 40 Inhalation Medication Nebulizer or equivalent filled with the threshold check solution. The threshold check solution consists of 13.5 milligrams of Bitrex in 100 ml of 5% salt (NaCl) solution in distilled water.
- DeVilbiss Model 40 Inhalation Medication Nebulizer or equivalent filled with the fit test solution. The fit test solution consists of 337.5 milligrams of Bitrex in 200 ml of 5% salt (NaCl) solution in warm water.
- A CPR mannequin that meets the American Heart Association requirements for instruction of BLS classes, similar in style to the Laerdal Little Anne (Part # 123-01050)

Fit testing will consist of two parts:

A) Sensitivity testing
   a. The subject dons the test enclosure without wearing a respirator
   b. Throughout this section the subject shall breathe through their mouth with their tongue slightly extended.
   c. The subject is instructed to report when they taste a bitter taste
   d. To produce the aerosol, the nebulizer bulb is firmly squeezed such that it collapses completely and then allowed to fully re-expand.
   e. An initial ten squeezes are repeated rapidly and then the test subject is asked whether the Bitrex can be tasted. If the test subject reports tasting the bitter taste during the ten squeezes, the screening test is completed. The taste threshold is noted as ten regardless of the number of squeezes actually completed.
   f. If the first response is negative, ten more squeezes are repeated rapidly and the test subject is again asked whether the Bitrex is tasted. If the test subject reports tasting the bitter taste during the second ten squeezes, the screening test is completed. The taste threshold is noted as twenty regardless of the number of squeezes actually completed.
   g. If the second response is negative, ten more squeezes are repeated rapidly and the test subject is again asked whether the Bitrex is tasted. If the test subject reports tasting the bitter taste during the third set of ten squeezes, the screening test is completed. The taste threshold is noted as thirty regardless of the number of squeezes actually completed.
   h. The investigator will take note of the number of squeezes required to solicit a taste response.
   i. If the Bitrex is not tasted after 30 squeezes (step 10), the test subject is unable to taste Bitrex and study procedures are terminated.
   j. If a taste response is elicited, the test subject shall be asked to take note of the taste for reference in the fit test.
   k. Correct use of the nebulizer means that approximately 1 ml of liquid is used at a time in the nebulizer body.
   l. The nebulizer shall be thoroughly rinsed in water, shaken to dry, and refilled at least each morning and afternoon or at least every four hours.

B) Simulated CPR
   a. The test subject may not eat, drink (except plain water), smoke, or chew gum for 15 minutes before the test.
   b. The test subject shall don the same enclosure from the threshold check procedure while wearing the study respirator. The respirator shall be properly adjusted and equipped with any type particulate filter(s).
   c. A second DeVilbiss Model 40 Inhalation Medication Nebulizer or equivalent is used to spray the fit test solution into the enclosure. This nebulizer shall be clearly marked to distinguish it from the screening test solution nebulizer.
   d. As before, the test subject shall breathe through his or her slightly open mouth with tongue extended, and be instructed to report if he/she tastes the bitter taste of Bitrex.
   e. The nebulizer is inserted into the hole in the front of the enclosure and an initial concentration of the fit test solution is sprayed into the enclosure using the same number of squeezes (either

10, 20 or 30 squeezes) based on the number of squeezes required to elicit a taste response as noted during the screening test.

f. After generating the aerosol, a timer will be started and the test subject shall be instructed to perform chest compressions for 2 minutes on the mannequin.
g. Every 30 seconds the aerosol concentration shall be replenished using one half the number of squeezes used initially (e.g., 5, 10 or 15).
h. When two minutes has elapsed or the subject indicates they taste Bitrex then the timer and simulated CPR will be stopped.
i. The investigator will record the elapsed time.
