# Supplementary material for: Effectiveness of Elastomeric Half-Mask Respirators vs N95 Filtering Facepiece Respirators During Simulated Resuscitation: A Nonrandomized Controlled Trial
Source: JAMA Netw Open. 2021 Mar 16;4(3):e211564. doi: 10.1001/jamanetworkopen.2021.1564 (PMC7967080; doi:10.1001/jamanetworkopen.2021.1564)
Supplement: Supplement 2. — Data Sharing Statement [file jamanetwopen-e211564-s002.pdf]

# Data Sharing Statement

Barros. Effectiveness of Elastomeric Half-Mask Respirators vs N95 Filtering Facepiece Respirators During Simulated Resuscitation. *JAMA Netw Open*. Published March 16, 2021.
doi:10.1001/jamanetworkopen.2021.1564

**Data**
**Data available:** Yes
**Data types:** Deidentified participant data, Data dictionary
**How to access data:** ajb5d@virginia.edu
**When available:** With publication

**Supporting Documents**
**Document types:** None

**Additional Information**
**Who can access the data:** Approved Researchers
**Types of analyses:** Any purpose
**Mechanisms of data availability:** Signed data use agreement
